# Effect of virtual reality technology on balance ability intervention in stroke patients: a systematic review and meta-analysis

**DOI:** 10.3389/fphys.2026.1861105

**Published:** 2026-07-31

**Authors:** Xianyou Cui, Junhua Tang, Yuxuan Ying, Meng Tao, Hong Sun, Hailong Jiao

**Affiliations:** 1 School of Physical Education, Zhejiang Guangsha Vocational and Technical University of Construction, Dongyang, China; 2 Department of Sports Medicine, Dongyang Red Cross Hospital, Dongyang, China; 3 School of Physical Education, Shihezi University, Shihezi, China; 4 School of Exercise and Health, Shanghai University of Sport, Shanghai, China; 5 Ministry of Language and Culture, Xinjiang Second Medical College, Karamay, China

**Keywords:** balance, lower limb, rehabilitation, stroke, virtual reality

## Abstract

**Objective:**

This study is aimed at the assessment of the efficacy of virtual reality (VR) technology in improving balance ability among patients with stroke and analyze the potential moderating effects of participant characteristics, training programs, and other factors on interventions.

**Methods:**

An exhaustive search for published papers up to February 2025 was conducted across five electronic databases to identify randomized controlled trials (RCTs) investigating the effects of VR on balance ability in stroke patients. A systematic review and meta-analysis were performed to synthesize effect sizes using the standardized mean difference (SMD, Hedges’g). Subgroup analyses were conducted to explore sources of heterogeneity.

**Results:**

Meta analyses identified four principal findings. (1) BBS: VR significantly improved scores across 14 studies (SMD = 0.55, 95% CI: 0.23–0.88, P < 0.05), with the VR+CT subgroup showing the largest effect (SMD = 0.89). (2) TUG: VR significantly enhanced performance in 17 studies (SMD = −0.29, 95% CI: −0.53 to −0.05, P < 0.05); the VR+CT subgroup was optimal (SMD = −0.66, I^2^ = 36%). Egger’s test indicated publication bias (P < 0.05), and trim-and-fill correction reduced the effect size. (3) FRT: VR showed no significant benefit in 4 studies (SMD = 0.36, 95% CI: −0.06 to 0.77, P > 0.05). (4) 10MWT: VR showed a borderline, non-significant positive trend in 6 studies (SMD = 0.37, 95% CI: 0.00–0.74, P = 0.05, I^2^ = 36%).

**Conclusion:**

VR intervention may improve selected balance-related outcomes in stroke patients, especially when combined with conventional rehabilitation. VR significantly enhances BBS and TUG outcomes, shows a borderline positive trend on 10MWT, but has no significant effect on FRT. With good safety and high compliance, VR is a feasible adjunct for stroke rehabilitation. The evidence certainty is moderate to low; future large−sample, high−quality RCTs are needed to verify long−term efficacy and support clinical application.

**Systematic review registration:**

https://www.crd.york.ac.uk/prospero/, identifier CRD420251266836.

## Background

Stroke has become a global public health burden and a primary cause of functional disability worldwide (Whitall, 2004). Amid accelerating population aging, urbanization and widespread exposure to cardiovascular risk factors, the global incidence of stroke has risen sharply (MacKay-Lyons et al., 2020). Motor dysfunction is the most prevalent sequela of stroke, leading approximately 80% of patients that develop hemiplegia. Notably, half of these patients experience persistent symptoms, which severely impair mobility and quality of life—balance and coordination deficits (Kong and Lee, 2014). Maintaining balance relies on the continuous, synchronous integration of sensory and cognitive information from multiple systems, including the vestibular, visual, proprioceptive, and cognitive systems. Damage caused by stroke in these systems impairs patients’ balance ability and balance-dependent functional activities (Iruthayarajah et al., 2017).

While conventional rehabilitation therapy can effectively reduce stroke-related disability and promote functional recovery, it primarily focuses on peripheral limb training and lacks direct targeting of central nervous system regulation (Ready, 1985). With advances in rehabilitation technology, virtual reality(VR) and augmented reality technologies leverage immersive multimedia to simulate real or imagined environments, integrating sensory-motor experiences such as vision, hearing, touch to replicate real-world scenarios and activities. These immersive virtual environments are suitable for balance training, as they provide continuous visual input that acts as sensory feedback for the vestibular system, facilitating the execution of functional movements (Gerards et al., 2023; Global and Global, 2025).

In recent years, VR technology has emerged as a valuable tool in motor rehabilitation for stroke patients and has been validated to effectively improve balance ability in populations with Parkinson’s disease, multiple sclerosis, cerebral palsy, and stroke. However, critical research gaps and unresolved controversies persist, highlighting the urgent need for systematic synthesis of evidence. First, intervention effects vary across primary studies: for example, Cho et al. and Kim et al. demonstrated that VR technology enhances lower limb motor function, balance, and walking ability in stroke patients (Kim et al., 2009a; Cho and Lee, 2014a), while Barcala et al. and Cho et al. reported no significant improvement in patient balance (Cho et al., 2012; Barcala et al., 2013). Additionally, controversy persists regarding the efficacy of VR for balance rehabilitation in stroke patients, compounded by a paucity of studies with small sample sizes. While previous systematic reviews have examined VR for post-stroke rehabilitation, most have focused broadly on motor function or walking ability, with limited focus on specific balance outcomes (BBS, TUG, FRT, 10MWT). Furthermore, few have incorporated an updated evidence base up to 2025, conducted dose–response analysis to optimize VR training parameters, performed detailed subgroup analyses to explore moderators, or applied GRADE assessment to evaluate evidence certainty. Therefore, this study conducts a systematic review and meta-analysis of trials investigating VR-based interventions for balance dysfunction in stroke patients, aiming to provide evidence-based support for clinical rehabilitation practice.

## Methods

### Search strategy

The exhaustive review and meta-analysis was conducted in accordance with the Preferred Reporting Items for Systematic Reviews and Meta-Analyses guidelines (Moher et al., 2009), which outline standardized processes for meta-analyses. The research question was: What is the intervention effect of VR technology on balance ability in stroke patients?

Literature searches were performed across five electronic databases: the Cochrane Library, PubMed, Web of Science, Embase, and China National Knowledge Infrastructure (CNKI) up to February 21,2025. For consistency in study quality and reporting standards, only English-language peer-reviewed publications were included in the final analysis, even though CNKI was searched. The Boolean search strategy was as follows: (“virtual reality” OR “virtual environment” OR “virtual rehabilitation” OR “virtual game” OR “virtual therapy” OR “VR”) AND (“stroke” OR “cerebrovascular accident” OR “ischemic stroke” OR “hemorrhagic stroke” OR “hemiplegia”) AND (“balance” OR “postural balance” OR “postural stability” OR “mobility”) AND (“randomized controlled trial” OR “RCT”). For original studies with missing or unclear data, attempts were made to contact the corresponding authors via email to request additional information. This study was registered prospectively on the PROSPERO international prospective register of systematic reviews (Registration Number: CRD420251266836) and adheres to the PRISMA statement.

### Inclusion and exclusion criteria

Inclusion Criteria: (1) Participants: Patients diagnosed with stroke according to WHO criteria; clinically stable, clear consciousness, no severe cognitive impairment (defined as Mini-Mental State Examination score ≥ 24), no severe visual or hearing impairment. Notably, this criterion was explicitly specified as an inclusion requirement for the present review; studies that did not report MMSE scores or included patients with MMSE < 24 were excluded during screening. No upper age limit was applied.; (2) Interventions: Virtual reality (VR)–based balance training, alone or combined with conventional rehabilitation; (3) Comparators: Conventional rehabilitation or routine care; (4) Outcome indicators: Studies must report at least one of the following balance- or motor-related outcome measures: Berg Balance Scale (BBS), Timed Up and Go (TUG) test, 10-Meter Walk Test (10MWT), or Functional Reach Test (FRT); (5) Study design: Randomized controlled trials (RCTs).

Exclusion Criteria: (1) Duplicate publications or overlapping data; (2) Non-original studies (e.g., review articles, meta-analyses, case reports); (3) Control groups consisting of participants without stroke (e.g., healthy controls, patients with other neurological disorders); (4) Unavailable full text or inaccessible original data. Risk of bias was not an exclusion criterion and was assessed *post hoc* for quality appraisal only; (5) Studies with outcomes unrelated to the target indicators, or studies where relevant quantitative data could not be extracted for meta-analysis.

### Literature screening and data extraction

**Figure 1:**
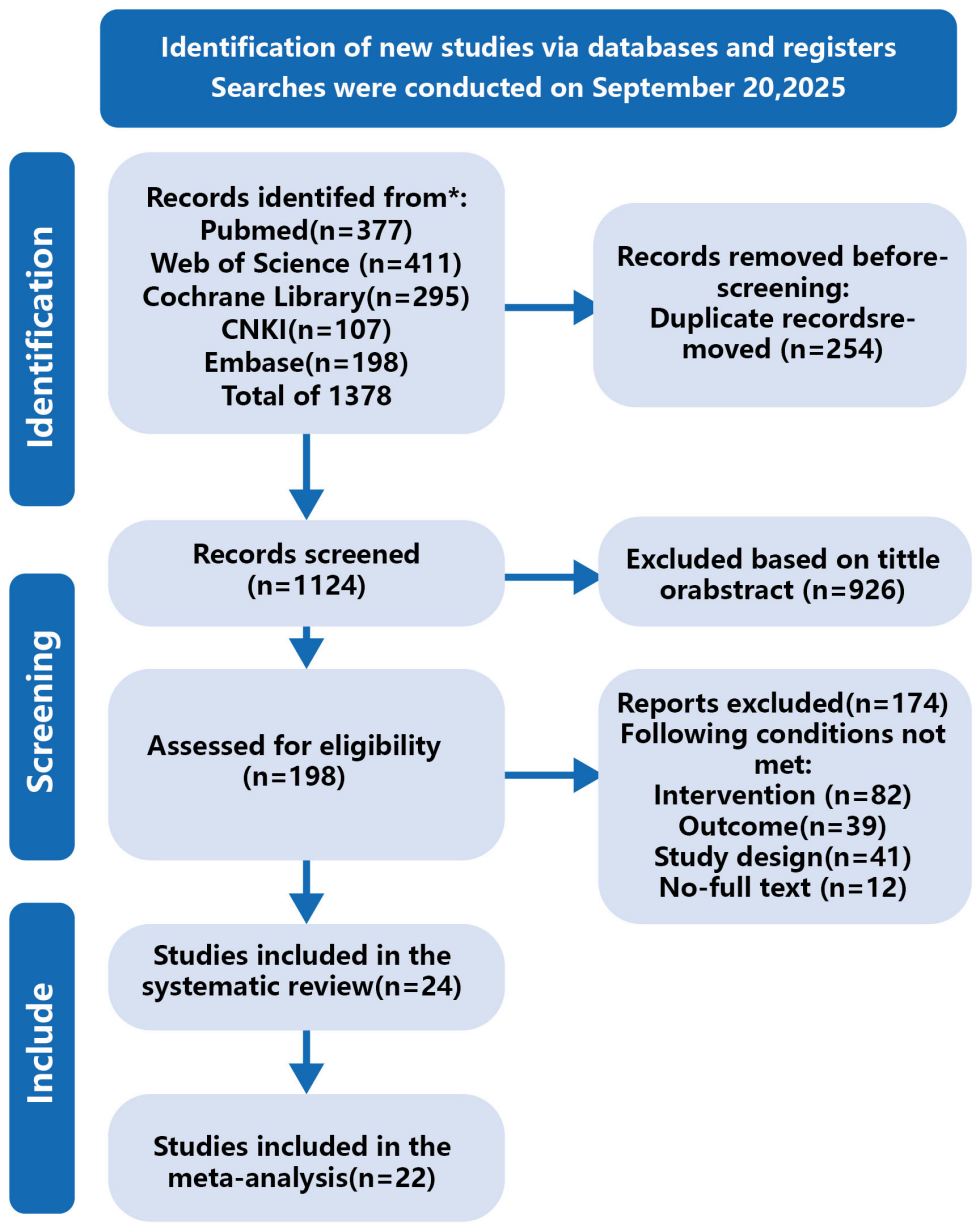
Flow chart of literature retrieval.

A total of 1,378 records were initially retrieved through database searches. After removing 254 duplicate records, 1,124 records proceeded to the preliminary screening phase. Following title and abstract screening, 37 articles were retained for full-text assessment. These articles were further evaluated against the predefined inclusion and exclusion criteria, with 22 studies ultimately meeting the eligibility requirements for inclusion in the systematic review and meta-analysis. The detailed literature screening process is illustrated in **Figure 1**.

Two independent researchers (Y.Y. and H.S.) conducted literature screening and data extraction, including: title and abstract review, full-text eligibility assessment, data extraction, and cross-validation. Disagreements between the two researchers were resolved through discussion; if consensus could not be reached, a third researcher (M.T.) was consulted to make the final decision. The extracted data included: first author and publication year, sample size, participant characteristics (e.g., age), intervention parameters (intervention modality, single-session duration, weekly frequency, and total intervention period), and all relevant outcome measures.

### Statistical analysis

Statistical analyses were performed using R software, Stata 16.0, and Review Manager 5.4. As the four key outcome indicators for assessing balance recovery in stroke patients recommended by international clinical practice guidelines, BBS, FRT, TUG and 10MWT were selected based on their wide recognition in clinical practice and research. The first two are core measures of static and dynamic balance, respectively, TUG reflects balance-related functional mobility and 10MWT evaluates gait-associated balance. The choice of effect size metric—MD or SMD—was determined by the consistency of measurement units across studies (Nagashima et al., 2019). In accordance with recommendations from the Cochrane Handbook for Systematic Reviews of Interventions; MD: Employed for outcomes measured with consistent scales such as identical measurement tools and units across studies; SMD: Adopted for outcomes assessed using different scales or tools for the same indicator. Hedges’ g was selected as the SMD estimator to correct for potential small-sample bias.

Heterogeneity among studies was evaluated using Cochran’s Q test (derived from Hedges’g), the I^2^ statistic, and 95% CI for the I^2^ value—these metrics quantify the extent of dispersion in true effects across studies (Nakagawa et al., 2017). I^2^ values were interpreted as follows: 0-25% (low heterogeneity), 25-50% (moderate heterogeneity), 50-75% (substantial heterogeneity), and 75-100% (considerable heterogeneity) (Cumpston et al., 2019). Subgroup analyses were carried out to examine potential moderating factors and explore sources of heterogeneity for key variables (including both categorical and continuous variables). A leave-one-out sensitivity analysis was performed to identify influential studies that might disproportionately affect the overall pooled results.

Publication bias was assessed using funnel plots (visual symmetry inspection) and Egger’s regression test. A P-value > 0.05 was considered indicative of no statistically significant publication bias (Egger et al., 1997).

### Dose-response analysis

We conducted a dose–response analysis to examine the relationship between VR training volume and balance improvement in stroke patients. To address differences in scale and direction of improvement across outcomes (BBS: higher = better; TUG/10MWT: lower = better; FRT: higher = better), all four balance outcomes were first standardized to a common effect-size metric (Hedges’ g). For TUG and 10MWT, the sign of the effect size was reversed so that higher values consistently indicated greater balance improvement across all measures. A unified balance improvement index was then calculated as the simple average of the four standardized, direction-aligned effect sizes. The robustness of this index was verified via sensitivity analyses. VR training dosage was calculated as session duration × weekly frequency × intervention weeks (Jiang et al., 2026). The RCS model was based on 22 independent data points (one per included study), and model stability was confirmed via leave-one-out sensitivity analysis.

Multiple regression models (linear, cubic polynomial, restricted cubic spline [RCS], and natural cubic spline) were tested, with RCS selected as the optimal model based on statistical fit and physiological plausibility (Harrell, 2001). Using likelihood ratio tests, a 3-knot RCS model was confirmed as superior. The apex of the inverted-U dose–response curve, representing the optimal VR dosage, was identified and validated via piecewise linear regression, with significance set at p < 0.05.

### Quality assessment

Using two complementary tools: the Cochrane Risk of Bias (RoB) 2.0 tool and the PEDro scale. The Cochrane RoB tool evaluates risk of bias across seven domains (randomization, allocation concealment, blinding, incomplete data, selective reporting, etc.), rated as low/unclear/high risk. A simplified A/B/C summary grade was added for overall quality description (A: low risk in all domains; B: mixed/unclear; C: high risk). The PEDro scale (0–11) provides a numeric quality score focusing on reporting rigor, blinding, and statistical reporting. Cochrane RoB focuses on bias risk; PEDro focuses on reporting and trial conduct. Both together provide a comprehensive quality profile. Disagreements were resolved by consensus or third reviewer (WD.C.), who provided a final judgment.

The assessment tool comprises seven domains, and each domain was rated by the evaluators as “low risk of bias,” “high risk of bias,” or “unclear risk of bias” (the standardized Cochrane rating categories). Study quality was graded based on the overall risk of bias: Grade A (low overall risk) if all seven domains met the “low risk of bias” criterion; Grade B (moderate overall risk) if partially met; and Grade C (high overall risk) if none of the domains met the “low risk of bias” criterion.

### Certainty of the evidence

Evidence of effectiveness for each study was combined with quality scores for use in discussing the results. The Grading of Recommendations Assessment, Development, and Evaluation(GRADE) methodology was used to rate the certainty of the evidence as “high,” “moderate,” “low,” or “very low” (Agarwal et al., 2022). GRADE was completed by two researchers, with differences resolved through consensus. This comprehensive assessment rates evidence as follows: (1) the risk of bias,downgraded by one level if “some concerns” and two levels if “high risk” of bias; (2) inconsistency, downgraded by one level when the impact of statistical heterogeneity (P) is moderate (>25%) and by two levels when high >75%; (3) imprecision: downgraded by one level when statistical power < 80% and if there was no clear direction of the effects; (4) risk of publication bias: downgrade one level if Egger’s test < 0.05.

## Results

### Research characteristics

A total of 1,378 records were initially retrieved through keyword searches. After excluding 1,355 records that failed to meet the predefined inclusion criteria (e.g., non-randomized controlled trials, irrelevant outcomes, incomplete data), 22 studies were ultimately included in the meta-analysis, involving 660 participants (Shown in **Table 1**).

**Table 1:** The characteristics of the studies included.

Author/year	N (IG/CG)		Age	Types	Time	Frequency	Period	Con	Indicators
(Anwar et al., 2021)	34	34	40.00-60.00	Wii-Fit Balance Board	60 min	3 times	6 weeks	CT	BBS
(Barcala et al., 2013)	20	20	51.37 ± 7.60	Wii-Fit Balance Board	30 min	5 times	8 weeks	CT	TUG;10MWT
(Cho et al., 2012)	11	11	65.26 ± 8.35	Wii-Fit Balance Board	30 min	3 times	6 weeks	CT	BBS;TUG
(Choi et al., 2018)	14	14	49.50 ± 10.21	Wii-Fit Balance Board	30 min	3 times	6 weeks	CT	BBS;TUG
(Gil-Gómez et al., 2011)	9	8	49.13 ± 21.18	Wii-Fit Balance Board	60 min	3–5 times	6 weeks	CT	BBS;TUG;10MWT
(Hung et al., 2014)	13	15	55.38 ± 9.95	Wii-Fit Balance Board	30 min	2 times	12 weeks	CT	TUG;FRT
(Lee et al., 2015)	12	12	49.16 ± 12.85	Wii-Fit Balance Board	30 min	3 times	6 weeks	CT	FRT
(Song and Park, 2015)	20	20	51.37 ± 7.60	Wii-Fit Balance Board	30 min	5 times	8 weeks	CT	TUG;10MWT
(Yatar and Yildirim, 2015)	15	15	62.80 ± 10.87	Wii-Fit Balance Board	30 min	3 times	4 weeks	CT	BBS;TUG;FRT
(Kim et al., 2009b)	12	12	52.42 ± 10.09	VR+CT	30 min	4 times	4 weeks	CT	BBS;10MWT
(Xu et al., 2025)	10	11	54.00 ± 11.90	VR+CT	30 min	3 times	4 weeks	CT	BBS;TUG
(Lee et al., 2016)	5	5	65.20 ± 5.00	VR+CT	30 min	3 times	4 weeks	CT	BBS;TUG;FRT
(Park et al., 2017)	10	10	62.00 ± 17.14	VR+CT	30 min	1 times	6 weeks	CT	BBS;TUG;10MWT
(Cho and Lee, 2014b)	15	15	65.86	VR+treadmill	30 min	3 times	6 weeks	CT	BBS;TUG
(Jung et al., 2012)	11	10	60.50 ± 8.60	VR+treadmill	30 min	5 times	3 weeks	CT	TUG
(Kang et al., 2012)	10	10	55.90 ± 6.50	VR+treadmill	30 min	3 times	4 weeks	CT	TUG;10MWT;FRT
(Kim and Lee, 2012)	9	9	47.44 ± 8.39	VR+treadmill	20 min	3 times	8 weeks	CT	BBS;TUG
(Bian et al., 2022)	23	21	53.25 ± 8.72	VR games	300 min	5 times	3 weeks	CT	BBS
(Kwak et al., 2024)	18	18	54.28 ± 17.74	VR games	30 min	3 times	5 weeks	CT	BBS;TUG
(Peláez-Vélez et al., 2023)	12	12	51.91 ± 18.58	VR games	15 min	3 times	6 weeks	CT	BBS
(Sana et al., 2023)	15	15	54.87 ± 9.47	Wii-Fit Balance Board	60 min	3 times	8 weeks	CT	TUG
(Yom et al., 2015)	10	10	mean age of 64.60	VR+CT	30 min	5 times	6 weeks	CT	TUG

N stands for sample size, Age of participants (years), CON control, IG intervention group, CG control group and CT conventional Training.Initial analysis.

In the included studies, participants’ age ranged from 58.9 to 77.32 years. The intervention modalities reported included: Wii-Fit Balance Board training, VR combined with conventional therapy (CT), VR combined with treadmill training, and VR-based gaming interventions. Intervention parameters varied across studies: single session duration ranged from 15 to 300 minutes, and total intervention period ranged from 3 to 12 weeks.

### Quality assessment

**Figure 2:**
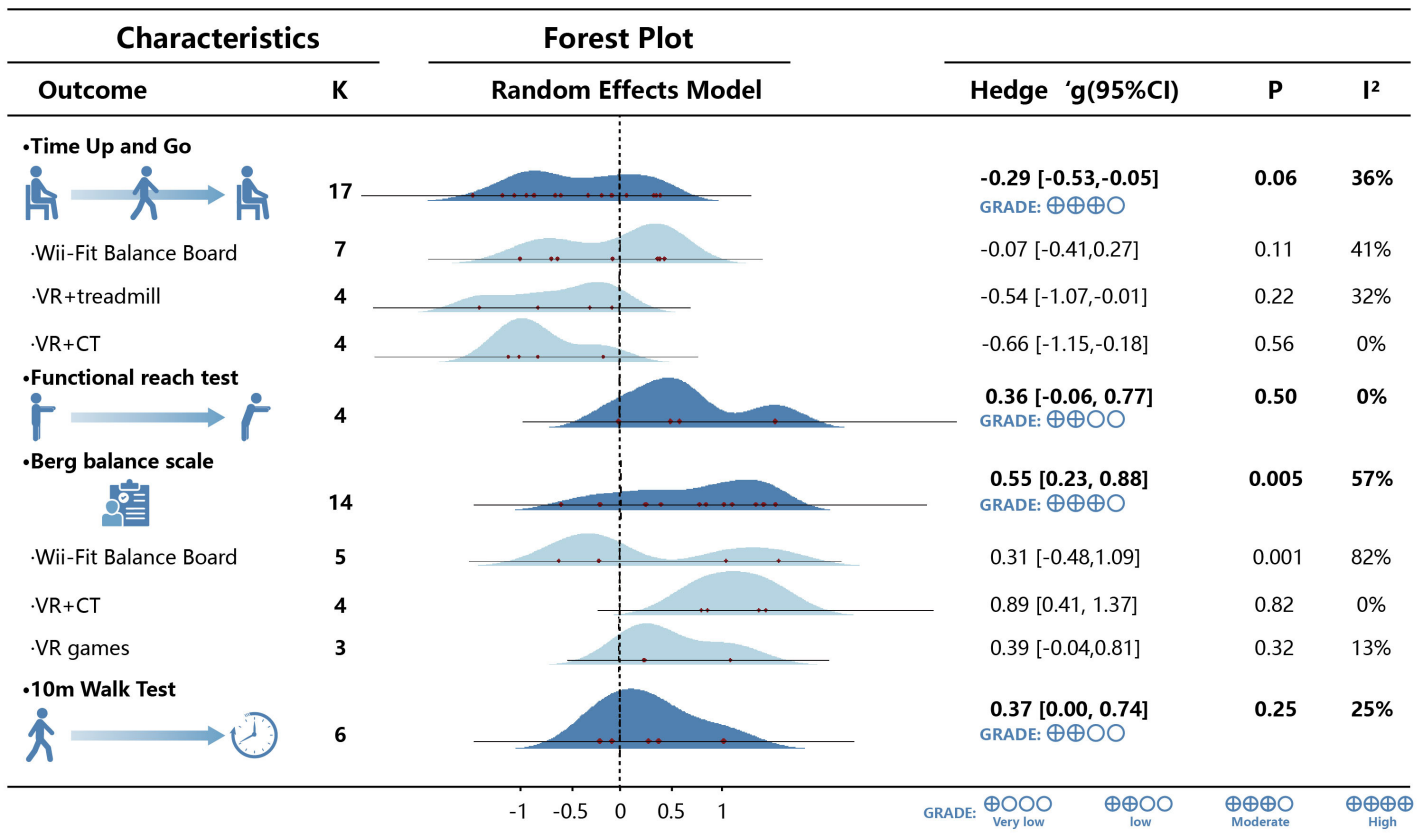
Effect of VR intervention on lower limb balance ability related index in stroke patients.

The quality of the 22 included RCTs was assessed using the Cochrane Risk of Bias tool, and Review Manager 5.4. Detailed results of the literature quality evaluation are presented in **Figure 2**, the evaluation focused on the following aspects: (1) Selection bias: whether the method of random sequence generation was used; (2) Allocation concealment: whether the allocation was effectively concealed; (3) Blinding: whether the subjects or investigators were blinded; (4) Data integrity: whether missing data was adequately reported and intention-to-treat analysis was applied; (5) Selective reporting: whether there was any selective reporting of outcomes; (6) Other biases: whether other factors contributed to potential bias.

As illustrated in **Figure 2**, the meta-analysis results for the impact of VR interventions on balance-related outcomes in stroke patients are as follows:

TUG test: Seventeen studies reported TUG scores. Pooled analysis showed that VR interventions significantly improved TUG scores (SMD = -0.29, 95% CI: -0.53 to -0.05, P < 0.05). Subgroup analysis revealed that the combined intervention of VR plus conventional therapy yielded the most pronounced effect (SMD = -0.66), with moderate heterogeneity observed across studies (I^2^ = 36%).

FRT: Four studies reported FRT scores. Pooled results indicated that VR interventions had no statistically significant effect on overall FRT scores (SMD = 0.36, 95% CI: -0.06 to 0.77, P > 0.05).

BBS: Fourteen studies reported BBS scores (note: higher BBS scores indicate better balance ability). Pooled analysis demonstrated that VR interventions significantly improved overall BBS scores (SMD = 0.55, 95% CI: 0.23 to 0.88, P 05). The most significant effect was observed in the subgroup receiving VR combined with conventional therapy (SMD = 0.89).

10MWT: Six studies reported 10MWT outcomes. Pooled results indicated a borderline positive trend without reaching statistical significance (SMD = 0.37, 95% CI: 0.00 to 0.74, P = 0.05), with moderate heterogeneity across studies (I^2^ = 36%).

### Secondary analysis

Subgroup analysis was performed to explore the modifying effects of various factors on lower limb balance ability in stroke patients. Forest plot results revealed moderate heterogeneity only for the TUG and BBS outcomes; thus, subgroup analysis was conducted specifically for these two indicators to identify the source of heterogeneity. However, no significant subgroup effects were observed with respect to age, intervention duration, intervention frequency, or treatment cycle (all P>0.05). In the case of the TUG subgroup analysis, the inter-subgroup heterogeneity test yielded no statistical significance (P>0.05), though the intervention cycle approached the threshold of statistical significance, suggesting a potential heterogeneity trend across different intervention cycle subgroups. The overall heterogeneity of TUG may be attributed to differences in intervention details among studies, such as the form of VR intervention (immersive vs. non-immersive) within this subgroup. Regarding the BBS subgroup analysis, no statistically significant inter-subgroup heterogeneity was detected (P>0.05). The overall heterogeneity of BBS is likely driven by variations in intervention details containing per-session training duration and specific training content, and patient baseline characteristics (e.g., disease severity) across different studies.

### Dose-response analysis of VR training

**Figure 3:**
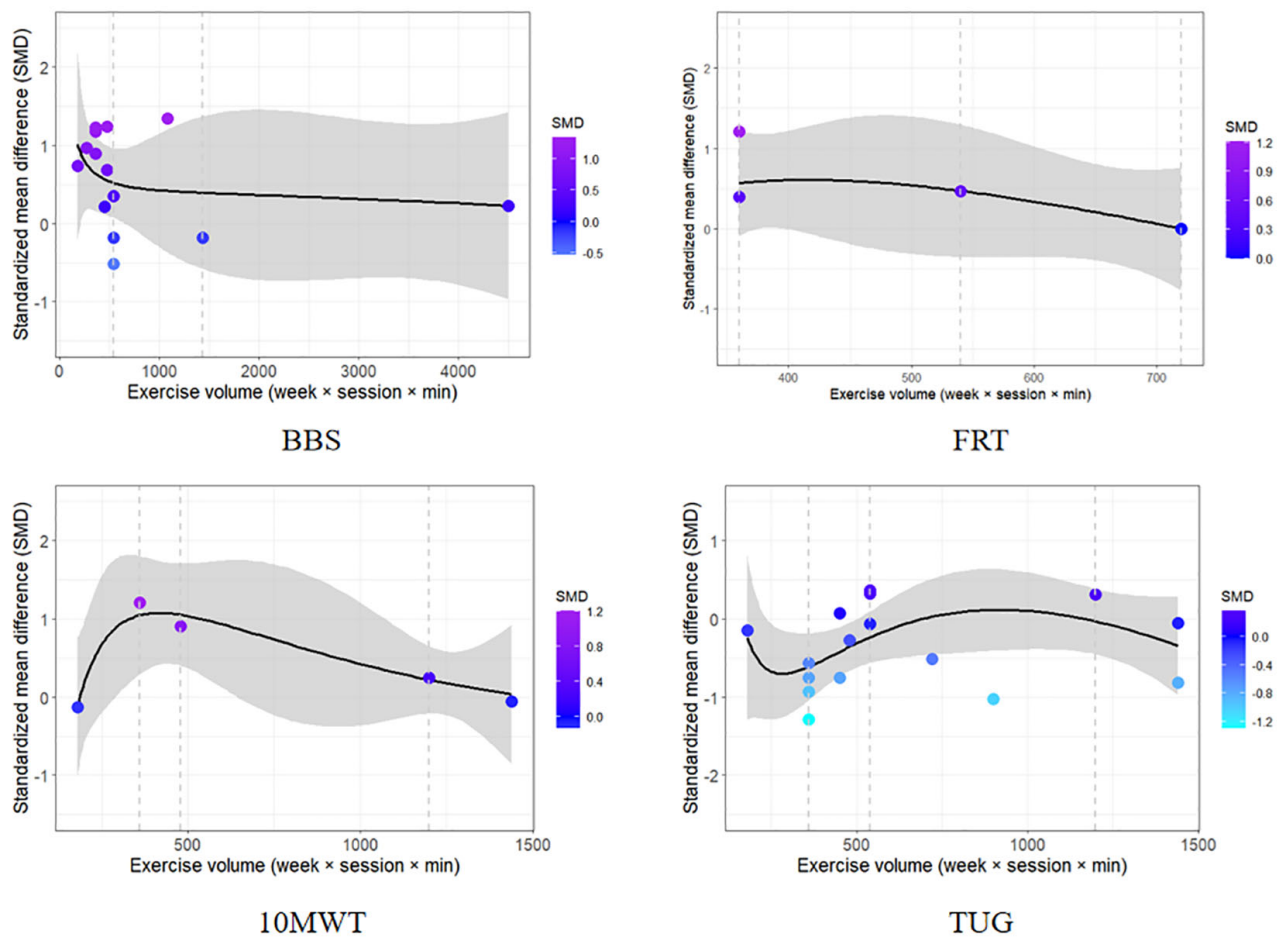
Dose–response relationships between VR training volume and balance outcomes in stroke patients.

RCS regression analyses identified non-linear dose–response relationships between VR training volume (week × session × min) and balance outcomes (**Figure 3**). BBS showed a stable positive effect across all volumes, while FRT demonstrated a gradual decline in benefit at higher volumes. Both 10MWT and TUG followed an inverted-U pattern, with peak improvements observed at moderate training volumes, suggesting that moderate VR doses may be optimal for maximizing balance benefits in stroke patients.

### Risk of bias and methodological quality

**Figure 4:**
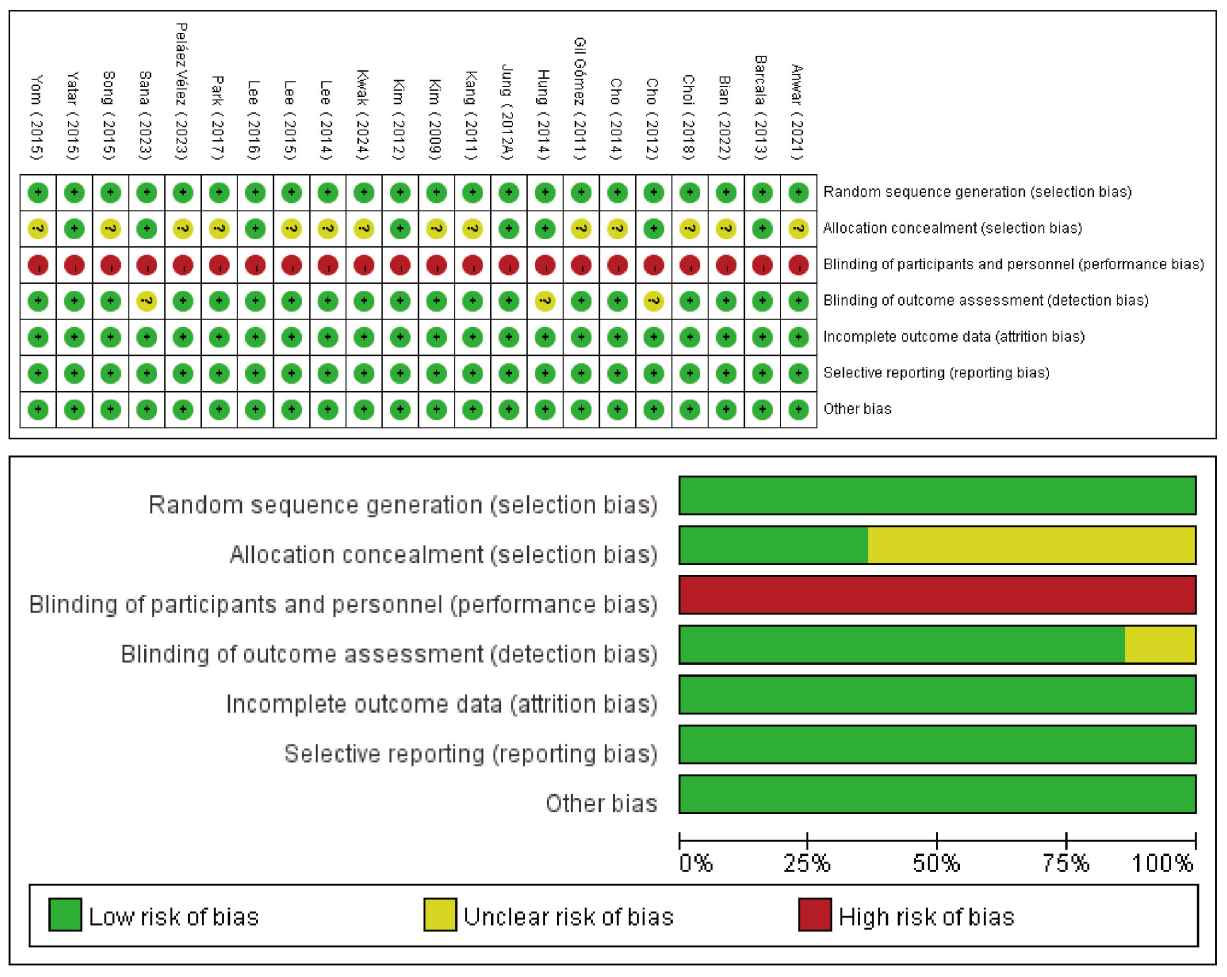
Risk of bias assessment diagram.

Risk of bias was assessed and reported for each included study (**Figure 4**). Overall, the included studies presented some concerns to high risk of bias, particularly in the domains of randomization and blinding. Specifically, no studies reported adequate allocation concealment, with 100% rated as “unclear risk”, thereby presenting concerns in the randomization process. Most critically, all studies were rated as high risk of bias for blinding of participants and personnel, as blinding is not feasible in non-pharmacological VR rehabilitation trials. In contrast, the risk of bias was low for incomplete outcome data, selective reporting, and other bias domains across all included trials. Blinding of outcome assessment was judged as low risk in most studies, with only a small proportion rated as unclear risk. Importantly, sensitivity analyses indicated that excluding studies at higher risk of bias did not significantly alter the main pooled results, supporting the robustness of our primary conclusions.

The PEDro scale (**Table 2**) assessment indicated that the overall methodological quality of the included studies was predominantly moderate, with a mean score of 6.3 out of 11. Most trials adequately defined eligibility criteria (D1), used random allocation (D2), achieved baseline comparability (D4), reported between-group statistical comparisons (D8), and provided point estimates and measures of variability (D10). However, key methodological limitations were evident, including the near-complete absence of blinding of participants, therapists, and outcome assessors (D5, D6), which is a well-recognized challenge in non-pharmacological exercise intervention studies. Additionally, intention-to-treat analyses (D7) were not consistently reported, with nearly half of the included trials failing to address incomplete outcome data, introducing a potential source of attrition bias. These factors underscore the need for cautious interpretation of the findings, although the overall methodological rigor was generally sufficient to support the primary conclusions of this meta-analysis.

**Table 2:** PEDro scale assessment results.

Author, year	D1	D2	D3	D4	D5	D6	D7	D8	D9	D10	D11	Total
(Lee et al., 2016)	Y	1	0	1	0	0	0	1	1	1	1	6
(Song and Park, 2015)	Y	1	0	1	0	0	0	1	1	1	1	6
(Yatar and Yildirim, 2015)	Y	1	0	1	0	0	0	1	1	1	1	6
(Anwar et al., 2021)	Y	1	0	1	0	0	1	1	1	1	1	7
(Barcala et al., 2013)	Y	1	0	1	0	0	0	1	1	1	1	6
(Cho et al., 2012)	Y	1	0	1	0	0	0	1	1	1	1	6
(Choi et al., 2018)	Y	1	0	1	0	0	1	1	1	1	1	7
(Gil-Gómez et al., 2011)	Y	1	0	1	0	0	1	1	1	1	1	7
(Hung et al., 2014)	Y	1	0	1	0	0	0	1	1	1	1	6
(Yom et al., 2015)	Y	1	0	1	0	0	1	1	1	1	1	6
(Bian et al., 2022)	Y	1	1	1	0	0	1	1	1	1	1	7
(Jung et al., 2012)	Y	1	0	1	0	0	1	1	1	1	1	6
(Kwak et al., 2024)	Y	1	0	1	0	0	1	1	1	1	1	6
(Peláez-Vélez et al., 2023)	Y	1	0	1	0	0	1	1	1	1	1	6
(Sana et al., 2023)	Y	1	1	1	0	0	1	1	1	1	1	7
(Kang et al., 2012)	Y	1	1	1	0	0	1	1	1	1	1	7
(Kim and Lee, 2012)	Y	1	0	1	0	0	0	1	1	1	1	5
(Cho and Lee, 2014b)	Y	1	1	1	0	0	1	1	1	1	1	7
(Kim and Lee, 2012)	Y	1	0	1	1	1	1	1	1	1	1	8
(Xu et al., 2025)	Y	1	0	1	0	0	1	1	1	1	1	6
(Lee et al., 2016)	Y	1	0	1	0	0	1	1	1	1	1	5
(Park et al., 2017)	Y	1	0	1	0	0	1	1	1	1	1	7

Studies scoring ≥6 are considered high quality, those scoring 4–5 moderate quality, and those scoring ≤3 low quality.

Eligibility criteria were specified (excluded in the total score). The subjects were randomly allocated to groups (in a crossover study, the subjects were randomly allocated in an order in which the treatments were received). Allocation was concealed. The groups were similar at baseline in terms of the most important prognostic indicators. All the subjects were blinded. All the therapists who administered the therapy were blinded. All assessors who measured at least one key outcome were blinded. Measures of at least one key outcome were obtained from more than 85% of the subjects initially allocated to groups. All subjects for whom outcome measures were available received the treatment or control condition as allocated or, where this was not the case, data for at least one key outcome were analyzed by “intention to treat”. the results of between-group statistical comparisons are reported for at least one key outcome. the study provides both point measures and measures of variability for at least one key outcome.

### Sensitivity analysis

Sensitivity analyses indicated that the pooled estimates for all outcomes remained stable, with no substantial changes observed upon the exclusion of any single study, This suggests that the overall results were not unduly influenced by any individual trial. Detailed findings from the sensitivity analyses are provided in Appendices.

### Publication bias analysis

Publication bias was evaluated using Egger’s regression test. No statistically significant publication bias was detected for BBS, FRT, and 10MWT. However, Egger’s regression test for the TUG test yielded a statistically significant result (P<0.05), suggesting potential publication bias for this indicator. To further investigate and adjust for this bias, a trim-and-fill analysis was conducted. To further investigate and adjust for this bias, a trim-and-fill analysis was conducted. After adjustment, the corrected pooled effect size for TUG was SMD = −0.21 (95% CI: −0.40 to −0.02, P < 0.05). The magnitude of the effect was attenuated but remained statistically significant, indicating that the original finding was robust to potential publication bias.

### Certainty of evidence

According to the GRADE framework, the certainty of evidence for each outcome ranged from low to moderate (**Table 3**.). Specifically, the evidence for the FRT and 10MWT was rated as low certainty, which reflects inherent limitations related to study methodological quality (e.g., high risk of bias in some included studies) and substantial heterogeneity among studies. For FRT (n=4 studies, total sample size=118) and 10MWT (n=6 studies, total sample size=156), statistical power calculated via G*Power 3.1 was <80%, and their 95% CIs crossed the null effect line (FRT: -0.06 to 0.77; 10MWT: 0.00 to 0.74), supporting the “Serious rating” for imprecision. In contrast, the evidence certainty for the TUG test and BBS was rated as moderate, indicating that while certain concerns (e.g., minor methodological flaws or moderate heterogeneity) were identified, confidence in the estimated intervention effects remains relatively high.

**Table 3:** GRADE of all outcomes.

Outcome	No of studies	Certainty assessment					Standardized mean effect (95% CI)	Grade*
		Risk of bias	Inconsistency	Indirectness	Imprecision	Other		
TUG	17 RCTs	Serious	Not serious	Not serious	Not serious	None	-0.29 [-0.53,-0.05]	⨁⨁⨁◯ MODERATE
FRT	4 RCTs	Serious	Serious	Not serious	Serious	None	0.36 [-0.06, 0.77]	⨁⨁◯◯ LOW
BBS	14 RCTs	Serious	Not serious	Not serious	Not serious	None	0.55 [0.23, 0.88]	⨁⨁⨁◯ MODERATE
10MWT	6 RCTs	Serious	Serious	Not serious	Serious	None	0.37 [0.00, 0.74]	⨁⨁◯◯ LOW

*Certainty of evidence according to Grading of Recommendations, Assessment, Development and Evaluations (GRADE):.

High: We are very confident in the estimated effect.

Moderate: Our confidence in the estimated effect is moderate.

Low: We have limited confidence in the estimated effect.

Very low: We have very little confidence in the estimated effect.

Overall, the evidence supporting the effects of the intervention on TUG and BBS outcomes is the most conclusive. In contrast, conclusions regarding FRT and 10MWT should be interpreted with caution due to the low certainty of the underlying evidence.

## Discussion

This systematic review and meta-analysis included 22 studies. Comprehensive pooled analysis revealed that VR-based therapy yielded more significant improvements in balance function compared to traditional therapy, with particularly robust effects observed for the TUG test and BBS. Adverse events were not a prespecified outcome in this review. No major adverse events were incidentally reported across the included studies, suggesting a generally acceptable safety profile, though this finding should be interpreted with caution. The overall quality of evidence from the meta-analysis was moderate (mean PEDro score = 6.3), though the majority of included studies had some concerns or high risk of bias—most notably in allocation concealment (55% of studies did not report adequate concealment), blinding (nearly all studies failed to blind participants or therapists due to the nature of VR interventions), and attrition (44% of studies had incomplete outcome data). These methodological limitations may lead to performance bias and selection bias, potentially overestimating the true effect of VR interventions.

Additionally, we compared different VR intervention modalities with conventional therapy (CT; defined as core muscle training, double/single bridge exercises, and center-of-gravity transfer training). The findings indicate that the combined intervention of VR plus CT exerted more significant effects on most balance indicators in stroke patients than either standalone VR or standalone CT. From a neurophysiological and clinical perspective, this synergistic superiority is mediated through multiple complementary mechanisms: First, CT establishes the base of foundational function by enhancing core muscle strength, improving joint range of motion, and restoring basic sensorimotor integration—addressing baseline deficits (e.g., weak trunk stability, insufficient muscle strength of lower limbs) that often limit the effectiveness of stand-alone VR training (Cech and Martin, 2023). Without this foundation, patients may struggle to complete VR tasks stably, leading to motor compensation or training interruption. Second, VR technology promotes neuroplasticity via immersive, task-specific scenarios. Real-time visual, auditory, and haptic feedback simulates real-world balance challenges, triggering the reorganization of cerebral cortex areas involved in balance control (e.g., primary motor cortex, cerebellum, and parietal lobe for sensory integration) (Hariharan, 2024). This reorganization enhances the cerebral power to dynamically adjust motor responses, a benefit rarely achieved by stand-alone CT which lacks immersive interactive feedback. Third, VR improves patient engagement and compliance through gamification and interactivity, reducing boredom associated with repetitive CT and ensuring consistent intervention implementation—critical for sustaining neuroplasticity and functional recovery (Emaliyawati et al., 2025). Fourth, the combination enables adaptive progression. CT provides standardized, low-to-moderate intensity training to build tolerance, while VR adjusts task difficulty based on real-time performance, ensuring gradual stimulation of balance function without excessive fatigue (Baur et al., 2018). Together, these mechanisms integrate the structural improvements from CT with the neuroplasticity and compliance benefits of VR, maximizing rehabilitation outcomes. This is likely due to standalone VR that can train balance control through virtual scenarios, it relies on patients having baseline muscle strength, joint range of motion, and trunk stability—functional deficits commonly observed in stroke patients that require prior improvement via CT (Fandim et al., 2021). Specifically, CT enhances the stability of the “spine-pelvis-hip joint” complex and improves muscle strength in key lower limb muscle groups (e.g., quadriceps femoris, anterior tibialis). This enhancement of foundational muscle strength enables patients to perform movements more stably in VR environments, reducing motor compensation and avoiding interrupted training due to insufficient muscle strength (Straathof et al., 2022). For the 10MWT, VR interventions showed only a marginal positive trend; the effect did not reach statistical significance. This trend may be attributed to the synergistic effects of VR combined with treadmill training: treadmills (a classic lower limb rehabilitation tool) provide standardized “gait rhythm guidance” via fixed speed and incline settings, helping stroke patients establish a stable “heel strike–toe off” gait pattern and compensating for the lack of “physical walking support” in standalone VR (Roerdink et al., 2011; Chang et al., 2021). In contrast, VR delivers real-time feedback on gait parameters (e.g., walking speed, step width, center-of-gravity deviation) and dynamically adjusts virtual scene difficulty (e.g., increasing road incline, adding obstacles), allowing patients to further optimize gait coordination and speed control on the basis of the stable gait rhythm established during treadmill training (Xie et al., 2026).

Furthermore, our dose–response analysis revealed nonlinear relationships between VR training volume and balance outcomes. The BBS exhibited a stable positive effect across most training volumes, suggesting consistent benefits for static balance within the applied dosage ranges. In contrast, FRT showed a gradual reduction in effect size with increasing training volume, implying that excessive VR training may not yield additional gains for functional reaching ability. Notably, both TUG and 10MWT followed an inverted−U pattern, with optimal improvements observed at moderate training volumes. These findings indicate that excessive VR training volume may lead to diminished rehabilitation returns, possibly due to cumulative fatigue, reduced task compliance, or overstimulation in stroke patients with limited physical tolerance. Therefore, moderate VR training doses appear to be most effective for enhancing balance−related mobility and gait function, which provides valuable evidence for clinical dosage standardization in stroke VR rehabilitation.

Gait function is a key determinant of lower limb balance ability (Gibbons et al., 2016). Compared to patients receiving traditional physical therapy, those undergoing VR-based therapy demonstrated more significant improvements in lower limb balance performance. We also systematically synthesized gait-related indicators from the included literature: minor differences were observed between VR and traditional therapy in terms of walking speed, step frequency, stride length, and step width, with trends consistently favoring VR. Obviously, improvements in walking speed were only statistically significant in the short term. Relevant studies have reported that CT and treadmill training are more beneficial for gait improvement during a one-month follow-up period (Corbetta et al., 2015).

As far as heterogeneity is concerned, subgroup analyses of age, intervention duration, frequency, and treatment cycle did not identify significant moderators, but moderate to substantial heterogeneity existed in BBS (I^2^=57%) and TUG (I^2^=36%). The heterogeneity unexplained may stem from unmeasured moderators related to VR intervention characteristics and patients’ baseline status: (1) Type of VR system: Included studies used diverse platforms (Wii-Fit Balance Board, immersive VR games, VR+treadmill), which vary in interactivity, feedback precision, and task complexity—factors that may differentially impact balance recovery. For example, fully immersive VR (e.g., head-mounted displays) provides stronger sensory immersion than non-immersive systems (e.g., Wii-Fit), possibly enhancing neuroplasticity (Ren et al., 2024). (2) Immersion level: Studies did not consistently report whether VR interventions were immersive (with isolated sensory input) or non-immersive (integrated with real-world environments), a distinction that may affect engagement and training effectiveness. (3) Stroke severity: Baseline balance function (e.g., BBS scores at enrollment) and stroke type (ischemic vs. hemorrhagic) were not uniformly reported, but these factors likely influence response to rehabilitation—patients with milder deficits may benefit more from VR’s interactive tasks, while those with severe impairments may require more foundational CT first. (4) Training content: VR tasks varied widely (e.g., balance games, obstacle avoidance, gait simulation), with no standardized protocol, leading to inconsistent stimulation of balance-related neural pathways.

Clinically, unexplained heterogeneity highlights the need for personalized VR rehabilitation: clinicians should consider VR system characteristics, patient tolerance for immersion, and baseline stroke severity when designing interventions. For example, non-immersive VR (e.g., Wii-Fit) may be more suitable for elderly patients or those with claustrophobia, while immersive VR could be prioritized for younger patients with mild to moderate deficits seeking higher engagement. Future studies should standardize reporting of these moderators to clarify their impact on outcomes. Notably, unexplained heterogeneity may also arise from differences in stroke stage (acute/subacute/chronic), baseline stroke severity, baseline balance function, type of VR system, intensity of comparator interventions, and specific VR training content, all of which may substantially influence intervention effects and interpretation.

### Limitations

This meta-analysis has several limitations that should be acknowledged. First, the methodological rigor of included studies was compromised by widespread concerns in allocation concealment, blinding, and attrition, which may lead the outcomes to bias and limit the generalizability of findings. Second, although we searched both English and Chinese databases (including CNKI), only English-language peer-reviewed publications were included in this review, which may introduce publication and language bias. Third, the number of eligible studies was relatively small (n=22), constraining statistical power—particularly for subgroup analyses (e.g., by patient gender, specific lower limb function outcome measures) and analyses of less commonly reported outcomes. Fourth, while VR intervention protocols varied across studies, most centered on general rehabilitation training; there was a notable paucity of studies incorporating specific aerobic or resistance training protocols. This limits conclusions regarding the interaction between VR and distinct exercise modalities. Fifth, subgroup analyses failed to identify significant moderators, and unexplained heterogeneity persisted, which may be due to unmeasured factors (e.g., VR immersion level, stroke severity) not captured in the included studies. At last, GRADE assessment of outcome measures revealed low certainty of evidence for two indicators (FRT and 10MWT), highlighting the need for further empirical investigation. Large-scale, high-quality randomized controlled trials with adequate sample sizes are warranted to validate these findings, particularly across diverse VR intervention modalities.

## Conclusion

Current evidence indicates that VR-based interventions targeting function of lower limbs in the patients with stroke are at least as effective as traditional physical therapy, with VR+CT yielding superior outcomes for BBS and TUG; evidence for 10MWT is limited to a borderline positive trend. Furthermore, VR offers distinct advantages: a favorable safety profile, enhanced patient engagement, and compatibility with diverse movement modalities—positioning it as a customizable adjunct or alternative to traditional therapeutic approaches. However, given the methodological limitations of included studies (notably in allocation concealment, blinding, and attrition) and the moderate-to-low certainty of evidence, recommendations should be tempered. VR rehabilitation should not replace gold-standard CT but rather be integrated into personalized plans, with implementation guided by basic function of patients, stroke severity, and tolerance for VR immersion. Current evidence suggests VR may offer a potential adjunctive approach to conventional rehabilitation for selected balance outcomes. However, evidence regarding long-term efficacy, feasibility, cost-effectiveness, adherence, and safety remains limited. Further high-quality trials are needed before wide-scale clinical implementation can be recommended.

## Data Availability

The original contributions presented in the study are included in the article/supplementary material. Further inquiries can be directed to the corresponding author.
